# Secretoglobin 3A2 Exhibits Anti-Fibrotic Activity in Bleomycin-Induced Pulmonary Fibrosis Model Mice

**DOI:** 10.1371/journal.pone.0142497

**Published:** 2015-11-11

**Authors:** Yan Cai, Shioko Kimura

**Affiliations:** Laboratory of Metabolism, National Cancer Institute, National Institutes of Health, Bethesda, Maryland, United States of America; French National Centre for Scientific Research, FRANCE

## Abstract

**Objective:**

Secretoglobin (SCGB) 3A2 is a novel lung-enriched cytokine, previously shown to exhibit anti-inflammatory, growth factor, and anti-fibrotic activities. The latter activity was demonstrated using exogenously-administered recombinant SCGB3A2 in the bleomycin (BLM)-induced pulmonary fibrosis model. Whether SCGB3A2 exhibits anti-fibrotic activity *in vivo* is not known.

**Methods:**

Mice null for the *Scgb3a2* gene were subjected to the BLM-induced pulmonary fibrosis model, and the severity of pulmonary fibrosis determined using histological and biochemical methods.

**Results:**

BLM treatment caused weight loss of both *Scgb3a2*-null and wild-type mice, however, the loss was far more pronounced in BLM-treated *Scgb3a2*-null than wild-type mice, and the weight of day 21 of BLM-treated *Scgb3a2*-null mice was about half of that of BLM-treated wild-type mice. Hematoxylin & Eosin, Masson Trichrome, and Sirius Red staining of lung sections, Ashcroft fibrosis scores, hydroxyproline contents, and the levels of mRNAs encoding various collagens demonstrated that BLM-treated *Scgb3a2*-null mouse lungs had more severe fibrosis than those of wild-type mouse lungs. Total and differential inflammatory cell numbers in bronchoalveolar lavage fluids, and levels of lung mRNAs including those encoding Th2 cytokines such as IL-4 and profibrotic cytokines such as TGFβ were higher in BLM-treated *Scgb3a2*-null mouse lungs as compared to those of wild-type mouse lungs. In contrast, mRNAs encoding surfactant proteins A, B, C, and D, and SCGB1A1 did not differ between BLM-treated *Scgb3a2*-null and wild-type mouse lungs.

**Conclusion:**

The role of SCGB3A2 in fibrosis was revisited using *Scgb3a2*-null mice and littermate controls in the BLM-induced pulmonary fibrosis model. The pulmonary fibrosis in the *Scgb3a2*-null mice was more severe than the wild-type controls, thus establishing that SCGB3A2 has anti-fibrotic activity *in vivo*. Importantly, surfactant proteins and SCGB1A1 appear not to be involved in the susceptibility of *Scgb3a2*-null mice to BLM-induced pulmonary fibrosis.

## Introduction

Secretoglobin (SCGB) 3A2, a new type of cytokine belonging to the SCGB gene superfamily, consists of secretory proteins of small molecular weight (~10 kDa) [1,2]. SCGB proteins are exclusively found in mammalian lineages, and are highly enriched in secretions such as lung, lacrimal gland, salivary gland, prostate and uterus. However, their biological functions are largely unknown [3]. The prototypical member of the SCGB gene superfamily, SCGB1A1, also called Club cell secretory protein (CCSP), Club cell 10-kDa protein (CC10), or Club cell 16-kDa protein (CC16), is believed have anti-inflammatory, anti-fibrotic, immunomodulatory, and tumor suppressive functions [4–8].

SCGB3A2 is predominantly expressed in epithelial cells of the trachea, bronchus, and bronchioles [2,9]. During lung development, SCGB3A2 expression is found at the growing tips of bronchi at embryonic day 11.5 of mouse gestation [10]. The expression level peaks around birth, and a half of the maximal level remains throughout adulthood [11]. This expression pattern suggested a role for SCGB3A2 in lung development. In fact, SCGB3A2 promoted embryonic lung development as demonstrated using *ex vivo* embryonic lung organ cultures in the presence of SCGB3A2, and *in vivo* by the administration of SCGB3A2 to pregnant female mice, followed by examination of pre-term pups [10]. In relation to this, SCGB3A2 was demonstrated to be an early Club cell marker in conjunction with Notch signaling [12].

Other biological roles for SCGB3A2 have also been reported. The anti-inflammatory activity of SCGB3A2 was originally suggested by the observation that *Scgb3a2* mRNA levels were reduced in the lungs of fungal-induced allergic inflammation model mice, which was restored by dexamethasone treatment [2], and the reduced levels of lung *Scgb3a2* mRNA were inversely correlated with increased levels of the pro-inflammatory cytokines interleukin (IL)-5 and IL-9 in bronchoalveolar lavage fluid (BALF) of the ovalbumin (OVA)-induced inflammation model mice [13,14]. Further, the anti-inflammatory activity of SCGB3A2 was demonstrated by the intranasal administration of recombinant adenovirus expressing SCGB3A2 to the OVA-induced inflammation model mouse, in which forced airway expression of SCGB3A2 suppressed OVA-induced airway inflammation [15]. A recent study using *Scgb3a2*-null mice in the OVA-induced inflammation model unequivocally demonstrated an anti-inflammatory role for SCGB3A2 [16].

Anti-fibrotic activity of SCGB3A2 was demonstrated using bleomycin (BLM)-induced pulmonary fibrosis mouse model, in conjunction with intravenous administration of SCGB3A2 [17,18]. The anti-fibrotic activity of SCGB3A2 was correlated with increased phosphorylation of STAT1 and increased levels of inhibitory SMAD7, which suppressed TGFβ signaling, resulting in reduced expression of various collagen genes and the development of fibrosis [17]. Both mouse and human recombinant SCGB3A2 proteins exhibited similar levels of anti-fibrotic activity when intravenously administered to mice in the BLM-induced pulmonary fibrosis model, suggesting that SCGB3A2 may be clinically relevant to treat pulmonary fibrosis in humans [18]. SCGB3A2 also serves as a marker for pulmonary carcinomas in mice and humans although the mechanism by which expression is enhanced in carcinomas is not known [19,20].

This study was initiated to revisit the anti-fibrotic role of SCGB3A2 using *Scgb3a2*-null mice in the BLM-induced pulmonary fibrosis model. *Scgb3a2*-null mice are more susceptible to BLM-induced pulmonary fibrosis, thus establishing that SCGB3A2 exhibits anti-fibrotic activity.

## Materials and Methods

### Animal studies

The *Scgb3a2*-null mice used in this study were produced at NCI as previously described [16] and backcrossed to C57BL/6NCr ten times. Wild-type littermates were used as controls. Mouse genotypes were determined by PCR using the following primer pairs: (forward) 5’-ATC CTC GGG GAA AAG TTC TG-3’ and (reverse) 5’-CTA AAA TCA GGG GCC AGA CA-3’ for knockout (KO) allele, and (forward) 5’-ACC GTC TCC CTG TTG TTG AC-3’ and (reverse) 5’-CAC GTA GCA AAG GCT TCT CC-3’ for wild-type (WT) allele. The PCR products were 362 bp and 227 bp for KO and WT alleles, respectively. All mice were housed in a temperature and humidity controlled specific pathogen-free facility under a 12 hour light/dark cycle with free access to water and conventional food. For the BLM-induced pulmonary fibrosis model, 8-wk-old male mice (25–28 g) were intratracheally intubated under anesthesia with ketamine (100 mg/kg)-xylazine (10 mg/kg) and dosed with BLM (1.2 U/kg, Sigma-Aldrich, B8416–15UN) or PBS as control at day 0. Mice were killed on day 21 by carbon dioxide asphyxiation from a bottle source, and bronchoalveolar lavage (BAL) fluids obtained by lavaging lungs with 1 ml PBS. The collected BAL fluids were used for counting inflammatory cell numbers with a hemocytometer. Differential cell count was carried out using cytospin preparations of BALF centrifuged through Shandon Cytofunnels (Thermo Fisher Scientific, Rockford, IL), followed by staining with Giemsa (Sigma-Aldrich, St. Louis, MO). Experiments were carried out using 5–6 mice per group, and were repeated more than 2 times. The health status of all animals was monitored daily. Mice with greater than 20% weight loss were supplemented by saline daily and closely monitored. If other humane endpoints deteriorated in addition to weight loss, they were immediately killed. The other human endpoints criteria were dyspnea (difficulties in breathing), hunched posture, rough hair coat, lethargy, persistent recumbence, or markedly reduced mobility, debilitating diarrhea, jaundice, anemia, significantly abnormal neurological signs, bleeding from any orifice, and self-induced trauma. All animal studies, including the conditions described above, were carried out after approval by the National Cancer Institute Animal Care and Use Committee.

### Histological analysis of lung sections

Lungs were inflated and fixed in 10% buffered formalin under 20-cm H_2_O pressure, embedded in paraffin, sectioned at 4 μm, and stained with hematoxylin and eosin (H&E). The sections were subjected to Masson Trichrome staining that detects collagen fibers. Fibrosis was quantified using the entire lung by the Ashcroft scoring system [21]. The degree of fibrosis was graded from 0 (normal lung) to 8 (severe distortion of structure, large fibrous areas, and honeycomb lesions). The mean score from all fields (magnification ×200, average 30 fields/animal) was taken as the fibrosis score. Sirius Red staining was carried out using Direct Red 80 (Sigma-Aldrich, #365548). The area of lung damage was assessed histologically by quantification of Sirus Red-positive areas using 10 fields (magnification, x40)/slide with Image J software. Scoring was carried out in blind fashion.

### Quantitation of hydroxyproline and cytokine levels

Hydroxyproline content was measured by using a hydroxyproline assay kit from Biovision (Milpitas, CA) according to the manufacturer's instructions with slight modification. In brief, whole lungs were homogenized in deionized H_2_O, using 100 μl H_2_O for every 10 mg of tissue. To a 100 μl of tissue homogenate, 200 μl of concentrated HCl (6 N) was added in a pressure-tight, Teflon-capped vial. The mixture was hydrolyzed at 120°C for 3 h, followed by filtration through a 45-μm syringe filter (Millipore, Bedford, MA), and 10 μl of hydrolyzed sample was transferred to a 96-well plate and evaporated to dryness under vacuum, then 100 μl chloramine-T reagent was added to each well. After incubation at room temperature for 5 min, 100 μl *p*-dimethylaminobenzaldehyde reagent was added to each well and incubation continued for 90 min at 60°C. Absorbance was measured at 560 nm in a microplate reader (SpectroMax Plus384, Molecular Devices, Sunnyvale, CA). The levels of mouse IL-4, IL-5, and IL-13 in BALF were determined by using ELISA kits from R&D systems (Mineapolis, MN) according to the manufacture’s protocol.

### qRT-PCR analysis

The whole left lobe of each lung was used for total RNA isolation with TRIzol, digested with DNase I, and reverse transcribed with Superscript II reverse transcriptase. Quantitative RT-PCR (qRT-PCR) was performed with an ABI Prism 7900 Sequence Detection System (Applied Biosystems, Foster City, CA) by using SYBR Green master mixture. The ΔΔCt method was used with 18S as normalization control. PCR conditions used were 50°C, 2 min; 95°C, 10 min; followed by 95°C, 15 sec; 60°C, 40 sec for 40 cycles with the following primers: *Col1a1* (forward) 5′-TAGGCCATTGTGTATGCAGC-3′, (reverse) 5′-ACATGTTCAGCTTTGTGGACC-3′; *Col3a1*(forward) 5′-TAGGACTGACCAAGGTGGCT-3′, (reverse) 5′-GGAACCTGGTTTCTTCTCACC-3′; *Col4a1* (forward) 5′-CACATTTTCCACAGCCAGAG-3′, (reverse) 5′-GTCTGGCTTCTGCTGCTCTT-3′; *Col12a1* (forward) 5′-TGAGGTCTGGGTAAAGGCAA-3′, (reverse) 5′-GTATGAGGTCACCGTCCAGG-3′; *Mmp12* (forward) 5′-TTTGGATTATTGGAATCTGC-3′, (reverse) 5′-ATGAGGCAGAAACGTGGACT-3′; *Il4* (forward) 5’-CGAGCTCACTCTCTGTGGTG-3’, (reverse) 5’-TGAACGAGGTCACAGGAGAA-3’; *Il5* (forward) 5’-CCCACGGACAGTTTGATTCT-3’, (reverse) 5’-GCAATGAGACGATGAGGCTT-3’;

*Il13* (forward) 5’-CACACTCCATACCATGCTGC-3’, (reverse) 5’-TGTGTCTCTCCCTCTGACCC-3’; *Il6* (forward) 5′- ACCAGAGGAAATTTTCAATAGGC-3′, (reverse) 5′-TGATGCACTTGCAGAAAACA-3′; *Cxcl13* (forward) 5′-TTGTGTAATGGGCTTCCAGA-3′, (reverse) 5′-AGGTTGAACTCCACCTCCAG-3′; *Ccl7* (forward) 5′-TTCCTCTTGGGGATCTTTTG-3′, (reverse) 5′-CTGCTTTCAGCATCCAAGTG-3′; *Tnfa* (forward) 5’-AGGGTCTGGGCCATAGAACT-3’, (reverse) 5’-CCACCACGCTCTTCTGTCTAC-3’; *Ctgf* (forward) 5’-GCTTGGCGATTTTAGGTGTC-3’, (reverse) 5’-CAGACTGGAGAAGCAGAGCC-3’; *Tgfb1* (forward) 5’-CAACCCAGGTCCTTCCTAAA-3’, (reverse) 5’-GGAGAGCCCTGGATACCAAC-3’;

*Acta2* (forward) 5’-GTTCAGTGGTGCCTCTGTCA-3’, (reverse) 5’-ACTGGGACGACATGGAAAAG-3’; *Sftpa* (forward) 5′-ACTCCCATTGTTTGCAGAATC-3′, (reverse) 5′-AAGGGAGAGCCTGGAGAAAG-3′; *Sftpb* (forward) 5′-ACAGCCAGCACACCCTTG-3′, (reverse) 5′-TTCTCTGAGCAACAGCTCCC-3′; *Sftpc* (forward) 5′-ATGAGAAGGCGTTTGAGGTG-3′, (reverse) 5′- AGCAAAGAGGTCCTGATGGA-3′; *Sftpd* (forward) 5′-GAGAGCCCCATAGGTCCTG-3′, (reverse) 5′- GTAGCCCAACAGAGAATGGC-3′; and 18S (forward) 5′-CGCGGTTCTATTTTGTTGGT-3′, (reverse) 5′-AGTCGGCATCGTTTATGGTC-3′.

### Statistical analysis

Statistical analysis was carried out by using the one-way ANOVA with Bonferroni correction (α = 0.05) for comparison among groups of animals, except for the analysis of weight loss data, in which Student's *t*-test was used. *P* < 0.05 was considered statistically significant.

## Results

### Subjection of *Scgb3a2-null* mice to BLM-induced pulmonary fibrosis model

**Fig 1:**
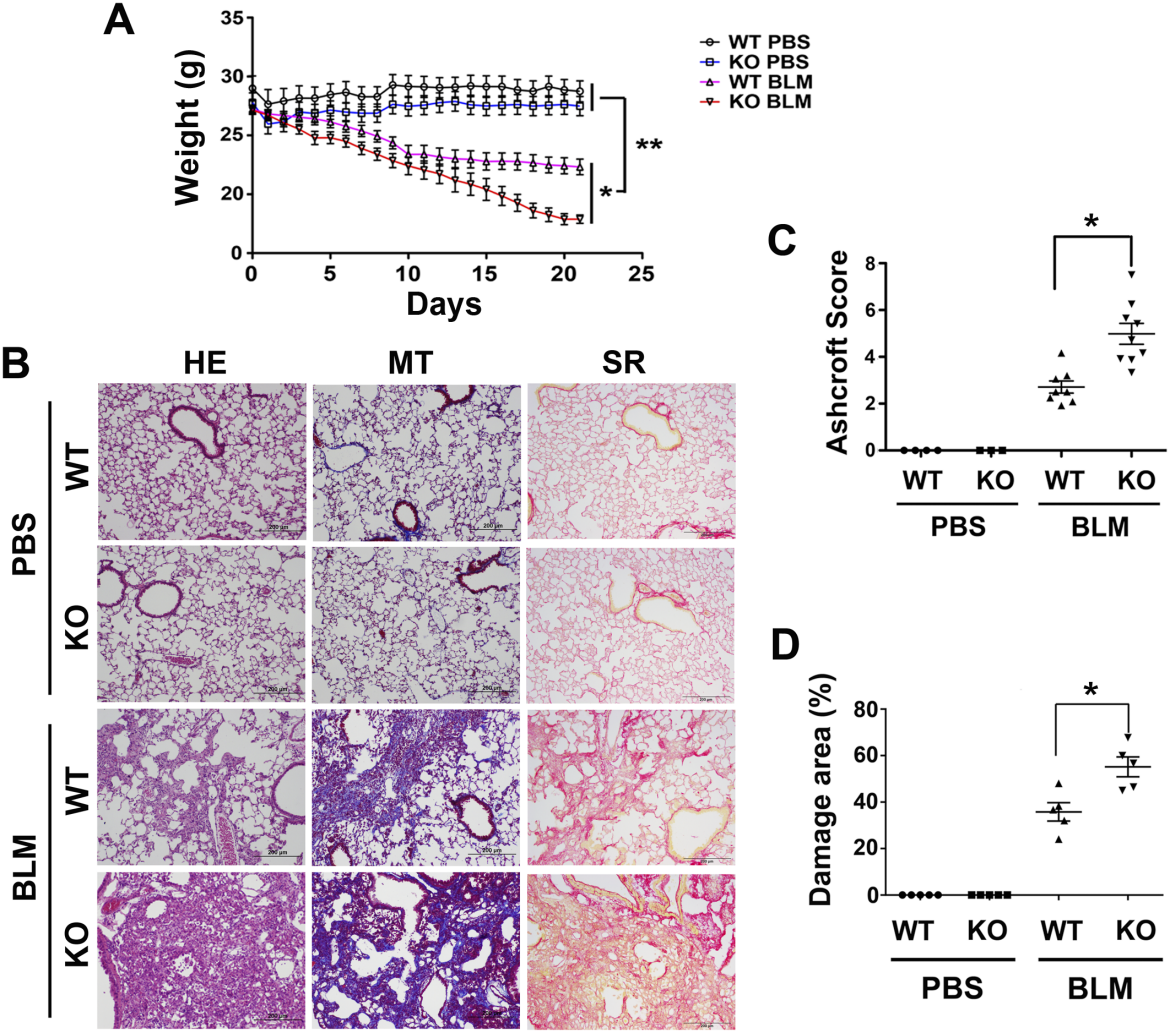
BLM-induced pulmonary fibrosis model. **(A)** Weight curves for wild-type (WT) and *Scgb3a2*-null (KO) mice during the entire experimental period of 21 days. Mice were intratracheally intubated bleomycin on day 0, and subjected to necropsy on day 21. Their weights were monitored daily. The mean ± SD are shown. N>10 in each group. *P<0.05, **P<0.01 by student’s *t*-test. **(B)** H&E staining (HE), Masson Trichrome staining that detects collagen fibers (MT), and Sirius Red staining for fibrosis severity (SR) of wild-type (WT) and *Scgb3a2*-null (KO) mice intubated with BLM or PBS as a control are shown. Representative images from N>3 in each group. Severe fibrosis is present in BLM-administered *Scgb3a2*-null lung. Scale bar: 200 μm. **(C)** Ashcroft Scores indicating the degree of fibrosis. **(D)** Damaged area determined by Sirius Red staining. The mean ± SD from N>3 in each group are shown. *P<0.05 by one-way ANOVA.

*Scgb3a2*-null and wild-type mice were intratracheally intubated with 1.2 U/kg of BLM or PBS as control at day 0 and their weights measured for 21 days until the mice were killed (Fig 1A). Weights of wild-type as well as *Scgb3a2*-null mice treated with PBS stayed at similar levels during the entire experimental period. In contrast, in the BLM-treated groups, *Scgb3a2*-null mice continuously lost weight right after BLM intubation, while wild-type controls started losing weights around day 5. The weight loss of BLM-treated wild-type mice decreased while BLM-treated *Scgb3a2*-null mice continued to lose weight. On the day of necropsy (day 21), the weights of *Scgb3a2*-null mice were about half the wild-type mice.

### Presence of severe fibrosis in *Scgb3a2*-null mouse lungs

On day 21, all mice were subjected to necropsy. Both *Scgb3a2*-null and wild-type mice treated with PBS showed normal lung histology while those treated with BLM demonstrated extensive fibrosis as demonstrated by H&E (Fig 1B, HE), Masson Trichrome staining that detects collagen fibers (Fig 1B, MT), and Sirius Red staining (Fig 1B, SR). The fibrosis severity was assessed by Ashcroft scoring, and lung damage was determined as a percentage of Sirius Red positive areas (Fig 1C and 1D). Both parameters were approximately twice higher in the lungs of BLM-treated *Scgb3a2*-null than BLM-treated wild-type mice.

### Increased collagens in BLM-treated *Scgb3a2*-null mouse lungs

**Fig 2:**
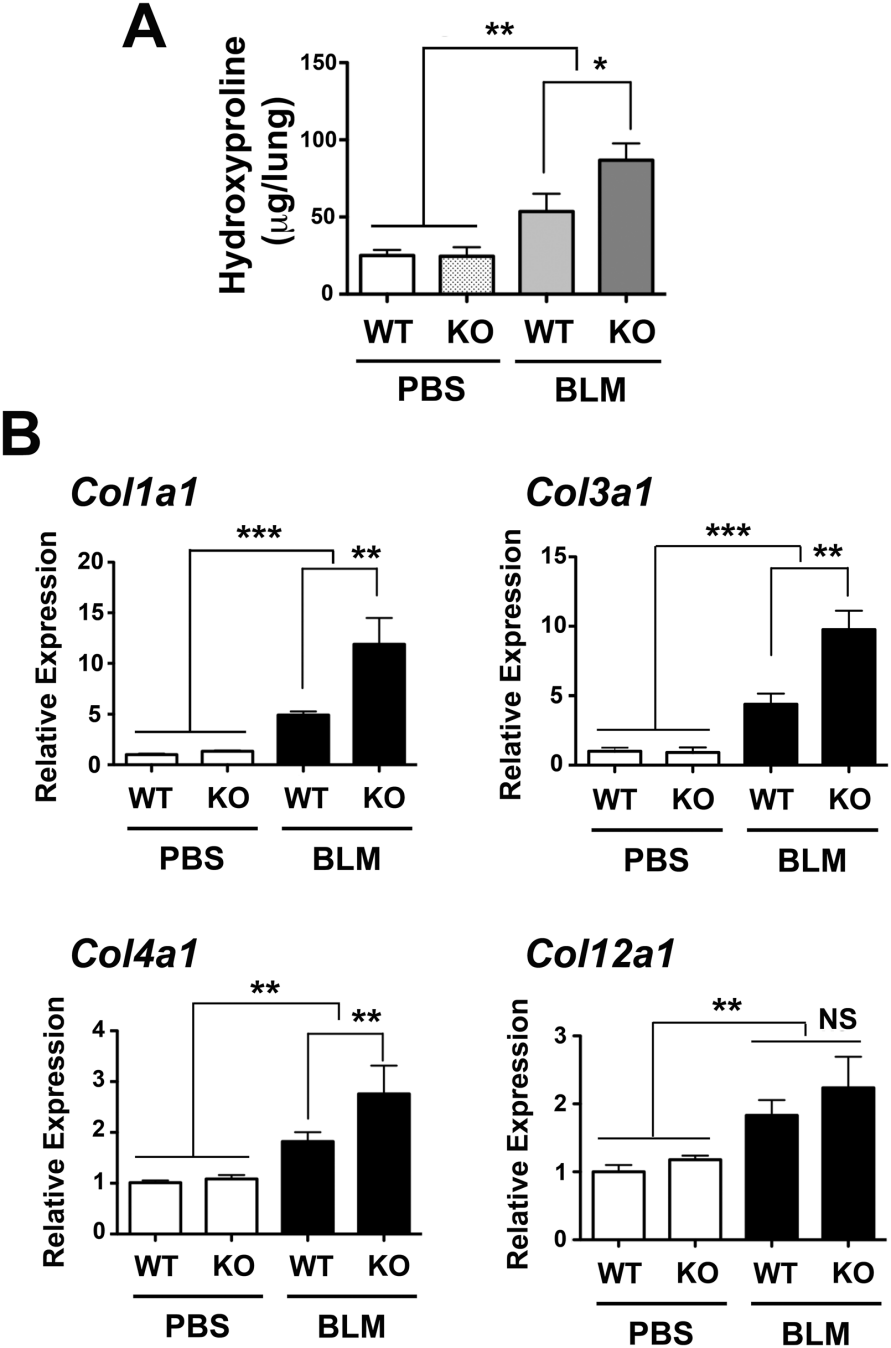
Increased collagens in BLM-treated *Scgb3a2*-null lungs. **(A)** Hydroxyproline content of lungs of wild-type (WT) and *Scgb3a2*-null (KO) mice intubated with BLM or PBS as control. The mean ± SD from N >6 in each group are shown. **(B)** qRT-PCR analysis of mRNAs for collagen 1a1 (*Col1a1*), collagen 3a1 (*Col3a1*), collagen 4a1 (*Col4a1*), and collagen 12a1 (*Col12a1*). Normalization control used was 18S. Relative expression levels with the mean ± SD from N>8 in each group are shown. *P<0.05, **P<0.01, and ***P<0.001 by one-way ANOVA. NS: not significant.

Both BLM-treated *Scgb3a2*-null as well as wild-type lungs had higher hydroxyproline, a major component of the collagen, than did the PBS-treated *Scgb3a2*-null and wild-type lungs (Fig 2A). Further, BLM-treated *Scgb3a2*-null lungs had approximately 1.6 fold higher hydroxyproline content than the lungs from BLM-treated wild-type mice. When levels of mRNAs encoding collagen 1A1, 3A1, 4A1, and 12A1 were measured by qRT-PCR, *Col1a1*, *Col 3a1*, and *Col 4a1* mRNAs were about twice higher in BLM-treated *Scgb3a2*-null lungs than BLM-treated wild-type lungs (Fig 2B). The levels of *Col12a1* mRNA were higher in BLM-treated groups of both wild-type and *Scgb3a2*-null lungs as compared with respective PBS control groups, however the levels were not different between BLM-treated *Scgb3a2*-null and wild-type lungs.

### Increased inflammation in BLM-treated *Scgb3a2*-null mouse lungs

**Fig 3:**
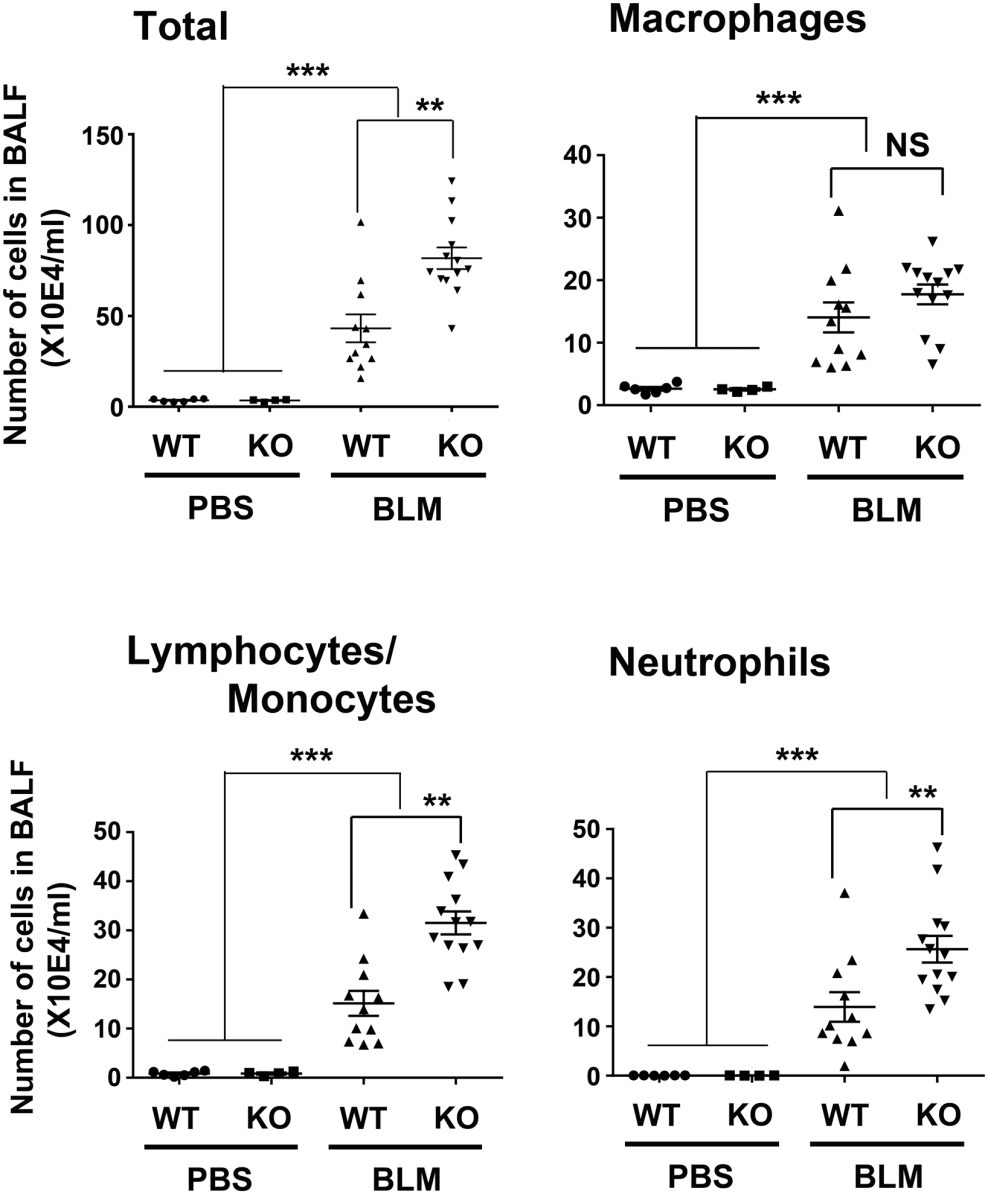
Increased inflammatory cells in BLM-treated *Scgb3a2*-null lungs. Total inflammatory cell numbers, macrophages, lymphocytes/monocytes, and neutrophils in BALF of wild-type (WT) and *Scgb3a2*-null (KO) mice intubated with BLM or PBS as control. The mean ± SD from N>5 in each group are shown. **P<0.01, and ***P<0.001 by one-way ANOVA. NS: not significant.

BALF total inflammatory cell number was counted revealing that both BLM-treated *Scgb3a2*-null mice as well as wild-type mice had extremely high numbers of inflammatory cells as compared with the PBS-treated groups (Fig 3). The *Scgb3a2*-null BALF had about two-fold more inflammatory cells, which were due to the increased number of lymphocytes/monocytes and neutrophils in their BALF than that of BLM-treated wild-type mice BALF. These results suggested that BLM-treated lungs had massive inflammation, and the severity of inflammation is higher in *Scgb3a2*-null than wild-type mice.

**Fig 4:**
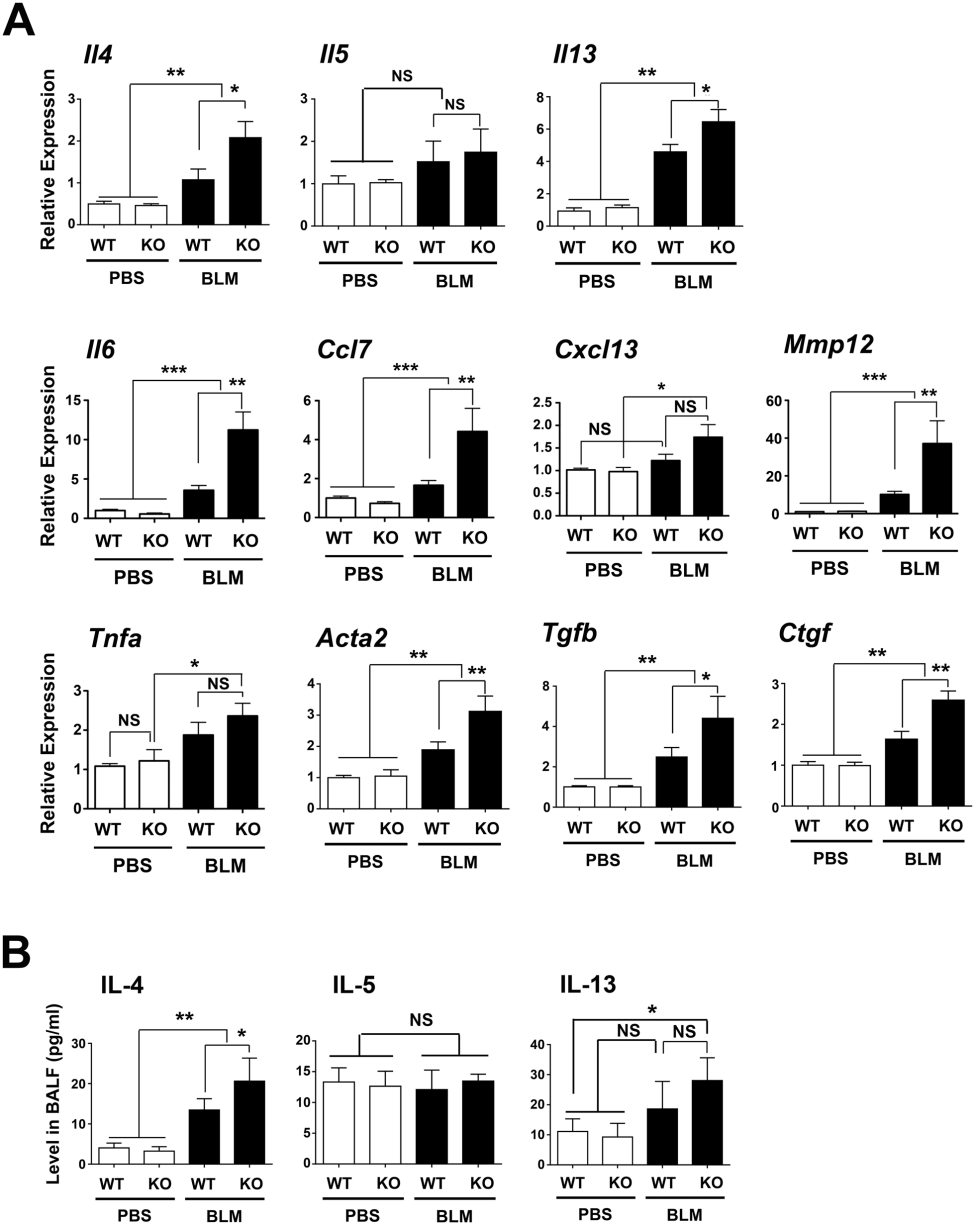
Increased cytokine levels in BLM-treated *Scgb3a2*-null lungs. **(A)** qRT-PCR analysis of mRNAs encoding IL-4, IL-5, IL-13, IL-6, CCL7, CXCL13, MMP12, TNFα, ACTA2 (α-smooth muscle actin), TGFβ, and CTGF in lungs of wild-type (WT) and *Scgb3a2*-null (KO) mice intubated with BLM or PBS as control. Normalization control used was 18S. **(B)** IL-4, IL-5, and IL-13 protein levels in BALF of wild-type (WT) and *Scgb3a2*-null (KO) mice intubated with BLM or PBS as control. Relative expression levels with the mean ± SD from N>8 in each group are shown. *P<0.05, **P<0.01, and ***P<0.001 by one-way ANOVA. NS: not significant.

The expression levels of mRNAs for several inflammatory cytokines were examined by qRT-PCR, including those encoding intgerleukin-4 (IL-4), IL-5, IL-6, IL-13, chemokine (C-C motif) ligand 7 (CCL7), chemokine (C-X-C motif) ligand 13 (CXCL13), matrix metalloproteinase 12 (MMP12), tumor necrosis factor α (TNFα), α-smooth muscle actin (ACTA2), connective tissue growth factor (CTGF), and transforming growth factor β (TGFβ) (Fig 4A) [22]. The expression of most mRNAs was higher in the BLM-treated groups of mice than the PBS-control mice. The expression of *Il4*, *Il13*, *Il6*, *Ccl7*, *Mmp12*, *Acta2*, *Tgfb*, and *Ctgf* was higher in lungs of BLM-treated *Scgb3a2*-null mice than BLM-treated wild-type mice. No differences were found for *Il5*, *Cxcl13*, and *Tnfa* mRNA levels between BLM-treated *Scgb3a2*-null and BLM-treated wild-type mouse lungs.

IL-4, IL-5 and IL-13 protein levels in BALF were determined using ELISA kit (Fig 4B). The levels of IL-4 were statistically significantly higher in lungs from mice treated with BLM than in the PBS controls, regardless of genotype, and in BLM-treated *Scgb3a2*-null lungs as compared with BLM-treated wild-type mouse lungs. IL-13 protein levels were higher in BLM-treated *Scgb3a2*-null lungs as compared with PBS-treated *Scgb3a2*-null and wild-type lungs with statistical significance. There were no statistically significant differences for IL-5 levels between PBS vs. BLM, and null vs. wild-type mice.

### No changes in expression of surfactant proteins in BLM-treated *Scgb3a2*-null mouse lungs

**Fig 5:**
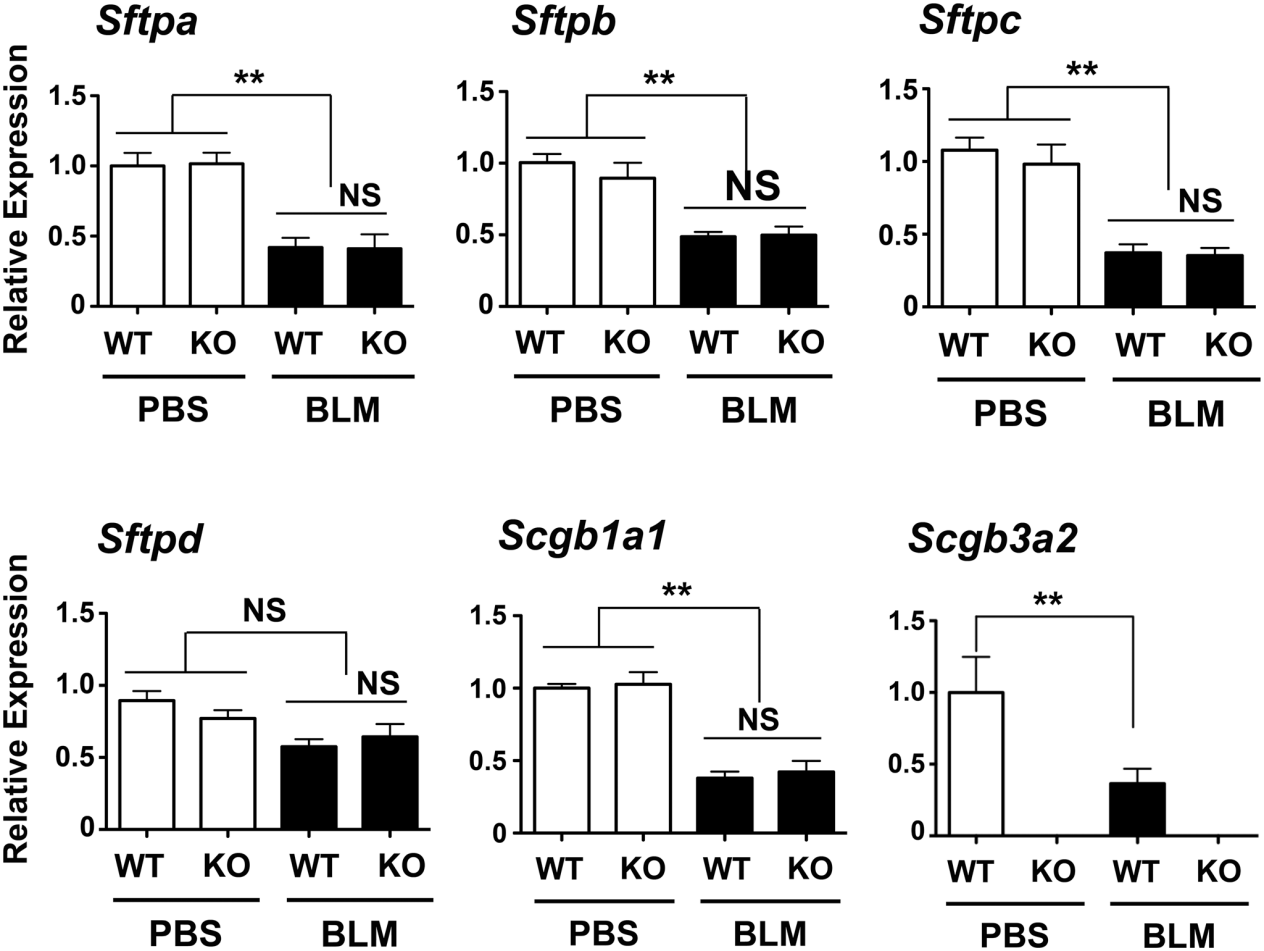
Analysis of mRNAs for surfactant proteins and SCGB proteins. qRT-PCR was carried out to determine levels of mRNAs encoding SPA (*Sftpa*), SP-B (S*ftpb*), SP-C (*Sftpc*), SP-D (*Sftpd*), *Scgb1a1* and *Scgb3a2* in the lungs of wild-type (WT) and *Scgb3a2*-null (KO) mice intubated with BLM or PBS as control. The normalization control was 18S. Relative expression levels with the mean ± SD from N>8 in each group are shown. **P<0.01 by one-way ANOVA. NS: not significant.

In order to examine whether BLM treatment causes differences in the expression levels of surfactant proteins, and whether the levels differ in the lungs of BLM-treated *Scgb3a2*-null mice compared to wild-type mice, the levels of mRNAs encoding surfactant proteins were examined by qRT-PCR (Fig 5). The expression of *Sftpa*, *Sftpb*, and *Sftpc* mRNAs was statistically significantly lower in BLM-treated group of mice than PBS control group for both mouse lines, while no differences were found between *Scgb3a2*-null and wild-type mice regardless of BLM treatment. The mRNA encoding SP-D stayed at similar levels in all groups. Since SCGB1A1 is known to play a role in fibrosis [5], the expression of *Scgb1a1* mRNA was also examined. Similarly, the *Scgb1a1* mRNA levels were lower in the BLM-treated groups than in the PBS control mice, while there was no difference observed between *Scgb3a2*-null and wild-type mice for both BLM treated and control groups. The lower level of *Scgb3a2* mRNA was noted in BLM-treated wild-type mouse lungs as compared with PBS-treated controls. These results demonstrated that BLM treatment reduced the expression of mRNAs for all the proteins examined except SP-D, and suggest that the severe fibrosis observed in the BLM-treated *Scgb3a2*-null was likely due to the lack of SCGB3A2 expression in this mouse line.

## Discussion

In this study, the role of SCGB3A2 was examined using *Scgb3a2*-null mice in the BLM-induced pulmonary fibrosis model. Previously, the anti-fibrotic activity of SCGB3A2 was demonstrated by exogenously administering SCGB3A2 to BLM-induced pulmonary fibrosis model mice [17,18]. In the BLM-treated *Scgb3a2*-null mice, more severe inflammation and fibrosis were demonstrated as compared with BLM-treated wild-type mice by all the parameters examined including histological analysis of lung tissues, collagen levels, hydroxyproline content, fibrosis severity, BALF inflammatory cells, and lung tissue inflammatory cytokine levels. The results clearly demonstrated that SCGB3A2 has anti-fibrotic activity.

SCGB1A1, the founding member of the SCGB gene superfamily was reported to possess anti-fibrotic activity when *Scgb1a1*-null mice were subjected to the BLM-induced pulmonary fibrosis model [5]. BLM-treated *Scgb1a1*-null mice had high mortality, expressed higher levels of pro-inflammatory cytokines such as IL-4 and IL-13, and the profibrotic cytokine TGFβ. These are very similar phenotypes to what was found for the *Scgb3a2*-null mice in the BLM-induced pulmonary fibrosis model. It was recently demonstrated that antiflammin-1, the carboxyl-terminal part of the third α-helix of SCGB1A1, has anti-inflammatory and anti-fibrotic activities in BLM-induced pulmonary fibrosis model [23]. Previous reports demonstrated that the levels of pro-inflammatory cytokines, IL-4, IL-5, and IL-13, and airway inflammation were suppressed in mice pre-treated with SCGB1A1 or SCGB3A2 in the OVA-induced allergic airway inflammation model [15,24]. BLM-induced pulmonary fibrosis involves lung inflammation and injury [25], and thus there is no surprise that the levels of the same pro-inflammatory cytokines such as IL-4 and IL-13 are altered in *Scgb1a1*-null and *Scgb3a2*-null mice in both the OVA-induced allergic airway inflammation and the BLM-induced pulmonary fibrosis models [15,24].

Previously SCGB1A1 was shown to bind to prostaglandin D_2_ (PGD_2_), resulting in the blockage of the PGD_2_ receptor (DP)-mediated nuclear factor-κB signaling through p38 mitogen-activated protein kinase, ERK1/2, and protein kinase C pathways in a cell type-specific manner, which plays a critical role in the inflammatory response [24]. Although the exact mechanism for the suppression of inflammation by SCGB3A2 is not known, it is possible that SCGB3A2 may also be involved in blocking of the DP-mediated nuclear factor-κB signaling. Further studies are required to address these questions.

Surfactant proteins are known to play a critical role in inflammation and homeostasis of lung [26–28]. Mice deficient for *Sftpc* showed increased and prolonged pulmonary fibrosis following intratracheal BLM administration [29]. In the current study, both *Scgb3a2*-null and wild-type mice have similarly decreased expression of mRNAs encoding for all surfactant proteins and SCGB1A1 after BLM treatment as compared with PBS control. The level of *Scgb3a2* mRNA was also decreased in BLM-treated wild-type mouse lungs as compared to PBS-treated lungs. It was previously reported that mRNA encoding surfactant proteins and SCGB1A1 decreased after BLM treatment with the greatest reduction at 2 weeks post-BLM [30]. However, of interest is that none of the mRNAs coding for surfactant proteins and SCGB1A1 have altered expression between *Scgb3a2*-null and wild-type mice after BLM treatment. There results suggest that the lack of SCGB3A2 expression, but not those of surfactant proteins and SCGB1A1 is likely to result in increased susceptibility of *Sgb3a2*-null mice to BLM-induced pulmonary fibrosis.

Based on previous studies of exogenous administration of SCGB3A2 to normal C57BL/6 mice [17,18] and the current study using *Scgb3a2*-null mice in the BLM-induced pulmonary fibrosis model, the levels of SCGB3A2 in the lung appears to be critical in suppressing pulmonary fibrosis regardless of whether it is given exogenously or being endogenously present *in vivo*. In patients with idiopathic pulmonary fibrosis (IPF), the level of SP-A in BALF is reduced and those having less than the median value of SP-A per total phospholipid for the cohort examined had shorter survival as compared to those with higher than the median value [31]. On the other hand, the association between increased serum SP-A levels and early mortality was reported among patients with IPF [32]. SP-D provides a useful serum marker for evaluating PF in patients with systemic sclerosis [33]. SCGB1A1 (CC16) can be used as a potential biomarker for PF in systemic sclerosis patients [34]. In a previous study, exogenously administered human recombinant SCGB3A2 showed a similar effect in suppressing BLM-induced pulmonary fibrosis using mouse model, suggesting that human SCGB3A2 most likely suppresses pulmonary fibrosis in humans [18]. These results suggest that an association may be present between endogenous SCGB3A2 levels in lung and the susceptibility to pulmonary fibrosis among human populations. Whether this is indeed the case requires further investigation.

## Conclusions

*Scgb3a2*-null mice are more susceptible to BLM-induced pulmonary fibrosis as compared to wild-type mice. Thus, SCGB3A2 has an anti-fibrotic activity, and potentially may be of value in the treatment of pulmonary fibrosis in humans.

## Supporting Information

S1 File ARRIVE checklist. (DOCX)
