# Supplementary material for: Secretoglobin 3A2 Exhibits Anti-Fibrotic Activity in Bleomycin-Induced Pulmonary Fibrosis Model Mice
Source: PLoS One. 2015 Nov 11;10(11):e0142497. doi: 10.1371/journal.pone.0142497 (PMC4641653; doi:10.1371/journal.pone.0142497)

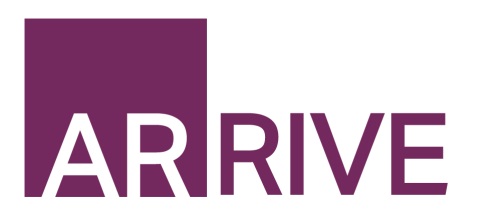


The ARRIVE Guidelines Checklist

Animal Research: Reporting In Vivo Experiments

Carol Kilkenny^1^, William J Browne^2^, Innes C Cuthill^3^, Michael Emerson^4^ and Douglas G Altman^5^

*^1^The National Centre for the Replacement, Refinement and Reduction of Animals in Research, London, UK, ^2^School of Veterinary Science, University of Bristol, Bristol, UK, ^3^School of Biological Sciences, University of Bristol, Bristol, UK, ^4^National Heart and Lung Institute, Imperial College London, UK, ^5^Centre for Statistics in Medicine, University of Oxford, Oxford, UK.*

|  | | ITEM | RECOMMENDATION | Section/ Paragraph |
| --- | --- | --- | --- | --- |
| 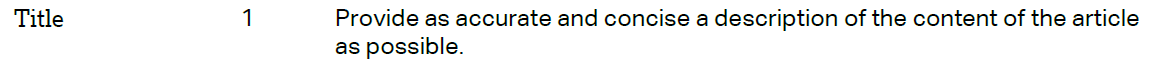 | | | Title |  |
| 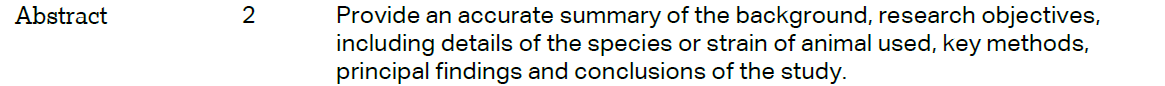 | | | Abstract |  |
| INTRODUCTION | | |  |  |
| 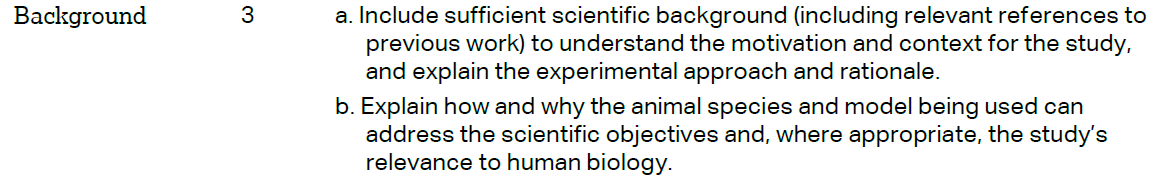 | | | Paragraph1-5  Paragraph2-5 |  |
| 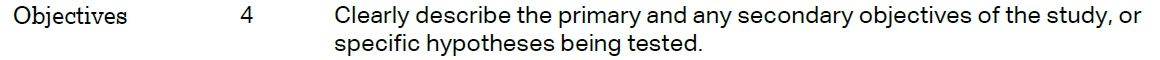 | | | Paragraph 5 |  |
| METHODS | | |  |  |
| 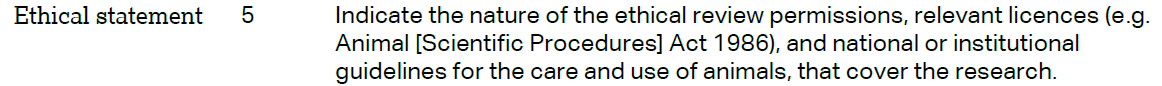 | | | Paragraph 1 |  |
| 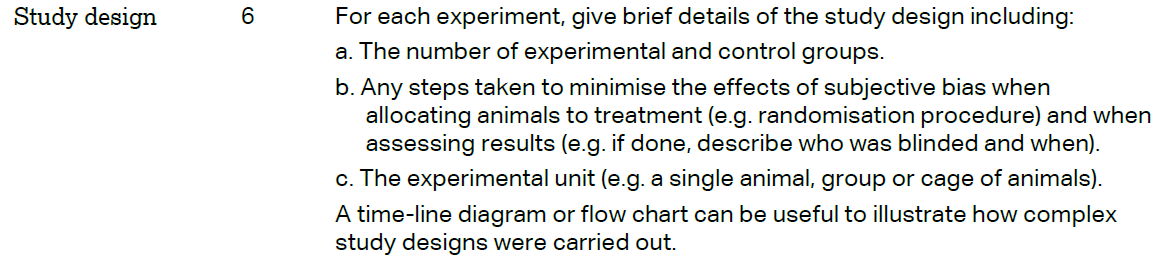 | | | Paragraph 1-2 |  |
| 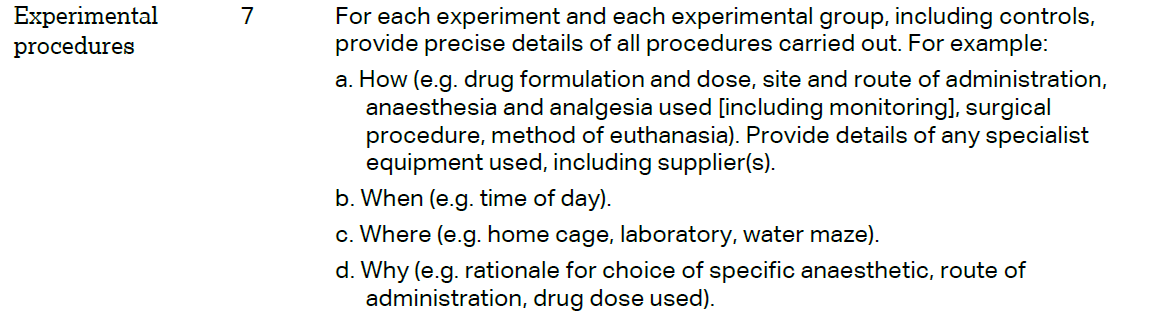 | | | Paragraph1 |  |
| 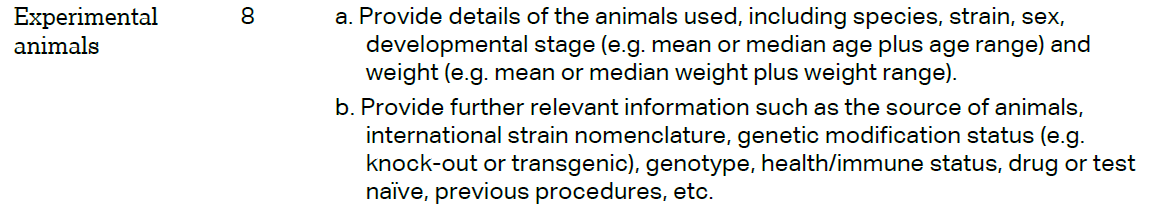 | | | Paragraph 1 |  |

The ARRIVE guidelines. Originally published in *PLoS Biology*, June 2010^1^

| 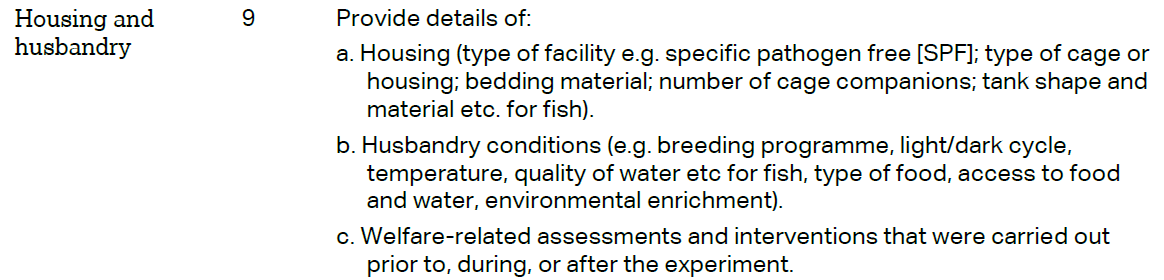 | Paragraph 1 | |
| --- | --- | --- |
| 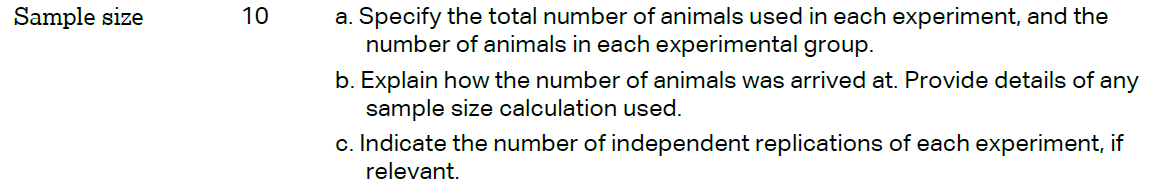 | Paragraph 1 | |
| 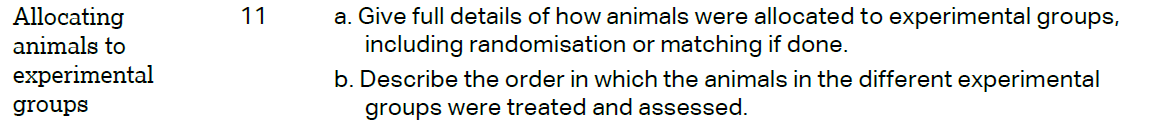 | Paragraph 1 | |
| 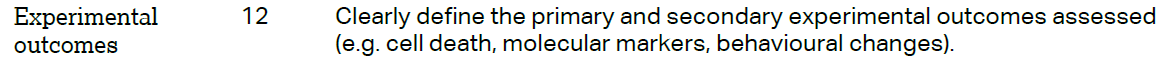 | Paragraph 1-4 | |
| 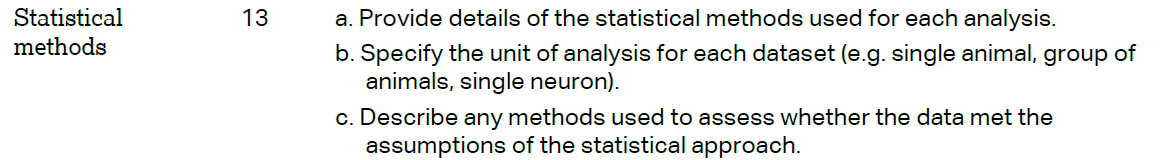 | Paragraph 5 | |
| RESULTS |  | |
| 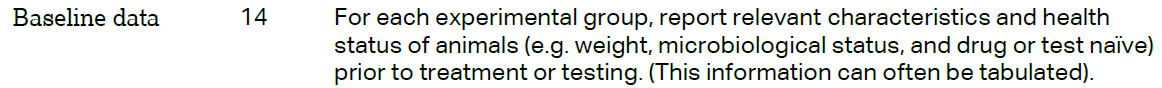 | Paragraph 1-5 | |
| 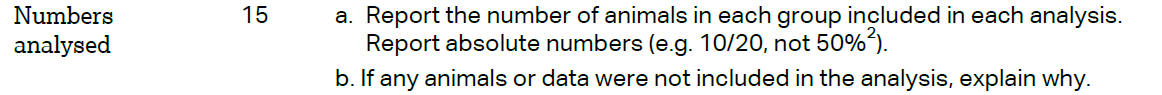 | Figure legends | |
| 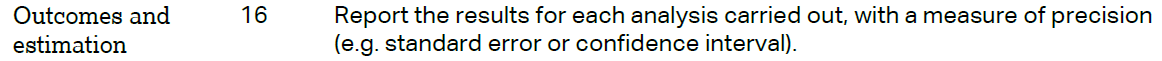 | Paragraph 1-5 | |
| 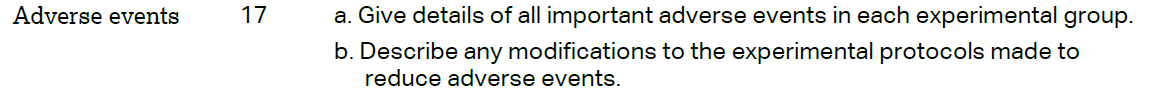 | Paragraph 1-5 | |
| DISCUSSION |  | |
| 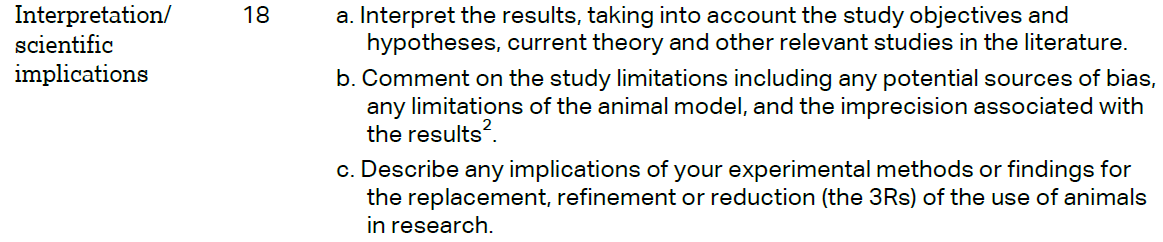 | Paragraph 1-3 | |
| 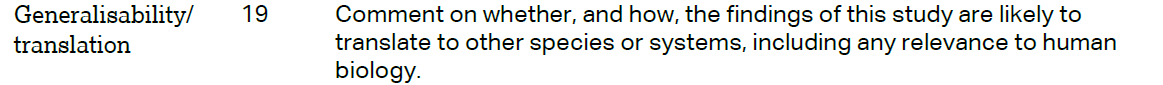 | Paragraph 3, Conclusions | |
| 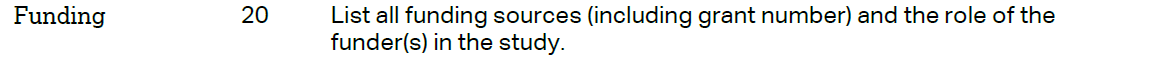 | | Funding section |


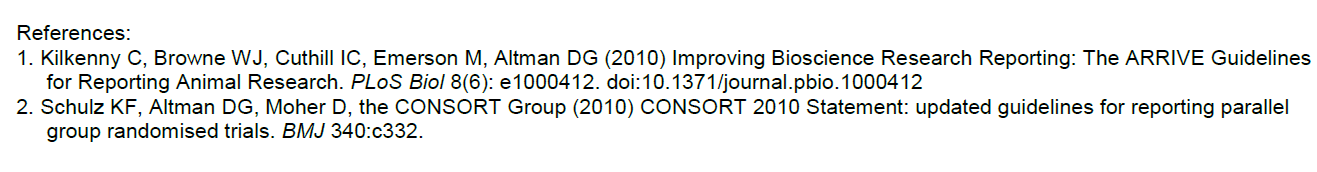

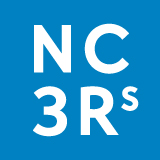

Supplement: S1 File — (DOCX) [file pone.0142497.s001.docx]
